# Determinants of Covid19 disease and of survival after Covid19 in MPN patients treated with ruxolitinib

**DOI:** 10.1038/s41408-023-00834-6

**Published:** 2023-05-03

**Authors:** Francesca Palandri, Elena M. Elli, Giuseppe Auteri, Massimiliano Bonifacio, Giulia Benevolo, Florian H. Heidel, Simona Paglia, Malgorzata M. Trawinska, Costanza Bosi, Elena Rossi, Mario Tiribelli, Alessia Tieghi, Alessandra Iurlo, Nicola Polverelli, Giovanni Caocci, Gianni Binotto, Francesco Cavazzini, Eloise Beggiato, Daniela Cilloni, Caterina Tatarelli, Francesco Mendicino, Maurizio Miglino, Monica Bocchia, Monica Crugnola, Camilla Mazzoni, Andrea D. Romagnoli, Giovanni Rindone, Sara Ceglie, Alessandra D’Addio, Eleonora Santoni, Daniele Cattaneo, Daniela Bartoletti, Roberto M. Lemoli, Mauro Krampera, Antonio Cuneo, Gianpietro C. Semenzato, Roberto Latagliata, Elisabetta Abruzzese, Nicola Vianelli, Michele Cavo, Alessandro Andriani, Valerio De Stefano, Giuseppe A. Palumbo, Massimo Breccia

**Affiliations:** 1 IRCCS Azienda Ospedaliero-Universitaria di Bologna, Istituto di Ematologia “Seràgnoli”, Bologna, Italy; 2 Hematology Division, San Gerardo Hospital, ASST Monza, Monza, Italy; 3 Dipartimento di Medicina Specialistica, Diagnostica e Sperimentale, Università di Bologna, Bologna, Italy; 4 Section of Hematology, Department of Medicine, University of Verona, Verona, Italy; 5 Hematology U, Department of Oncology, Città della Salute e della Scienza, Turin, Italy; 6 Innere Medicine C, Universitätsmedizin Greifswald, Greifswald, Germany; 7 Hematology, S.Eugenio Hospital, Tor Vergata University, ASL Roma2, Rome, Italy; 8 Division of Hematology, AUSL di Piacenza, Piacenza, Italy; 9 Section of Hematology, Department of Radiological and Hematological Sciences, Catholic University School of Medicine, Rome, Italy; 10 Fondazione Policlinico Universitario A. Gemelli IRCCS, Rome, Italy; 11 Division of Hematology and BMT, Azienda Sanitaria Universitaria Integrata di Udine, Udine, Italy; 12 Department of Hematology, Azienda USL - IRCCS di Reggio Emilia, Reggio Emilia, Italy; 13 Hematology Division, Foundation IRCCS Ca’ Granda Ospedale Maggiore Policlinico, Milan, Italy; 14 Unit of Blood Diseases and Stem Cell Transplantation, ASST Spedali Civili di Brescia, Brescia, Italy; 15 Hematology Unit, Businco Hospital, Department of Medical Sciences and Public Health, University of Cagliari, Cagliari, Italy; 16 Unit of Hematology and Clinical Immunology, University of Padova, Padova, Italy; 17 Division of Hematology, University of Ferrara, Ferrara, Italy; 18 Haematology Division, Department of Clinical and Biological Sciences, Ospedale San Luigi di Orbassano, University of Turin, Orbassano, Italy; 19 Hematology, S.Andrea Hospital, Rome, Italy; 20 Unit of Hematology, Hospital of Cosenza, Cosenza, Italy; 21 IRCCS Policlinico San Martino, Genova, Italy; 22 Dipartimento di Medicina interna e Specialità mediche, Università di Genova, Genova, Italy; 23 Hematology Unit, Azienda Ospedaliera Universitaria Senese, University of Siena, Siena, Italy; 24 Division of Hematology, Azienda Ospedaliero-Universitaria di Parma, Parma, Italy; 25 Division of Hematology, Onco-hematologic Department, AUSL della Romagna, Ravenna, Italy; 26 Hematology Unit, Ospedale Belcolle, Viterbo, Italy; 27 Villa Betania Hospital, Roma, Italy; 28 Dipartimento di Scienze Mediche, Chirurgiche e Tecnologie Avanzate “G.F. Ingrassia”, Università degli Studi di Catania, Catania, Italy; 29 A.O.U. Policlinico Umberto I, Università degli Studi di Roma “La Sapienza”, Rome, Italy

**Keywords:** Myeloproliferative disease, Clinical trials

## Introduction

The coronavirus disease 2019 (Covid19) pandemic caused by the spreading of the coronavirus SARS-CoV-2 has led to substantial mortality in patients with hematological diseases [1]. During the first wave of pandemic, patients with Philadelphia-negative chronic myeloproliferative neoplasms (MPN) including essential thrombocythemia (ET), polycythemia vera (PV), and myelofibrosis (MF) were reported at higher risk of acquiring SARS-CoV-2 and of having a poor outcome after infection, with a mortality rate of about 30%, increasing to 48% in MF patients [2].

Ruxolitinib is a *JAK1/2* inhibitor that is widely used both in MF and PV [3]. It may affect immunological response by decreasing the production of pro-inflammatory cytokines and by altering the function of several immune cells, including macrophages and B/T-lymphocytes [4]. Its use and discontinuation have been identified as risk factors for SARS-CoV-2 infection and Covid19-related death [5] Additionally, ruxolitinib-treated patients show lower serological response to anti-SARS-CoV-2 vaccination [6, 7].

Previous studies on Covid19 in MPN patients have included patients regardless of treatment type, with few patients treated with ruxolitinib at the time of the pandemic. Here, we explored features associated with Covid19 disease and survival after Covid19 in a large cohort of ruxolitinib-treated PV and MF patients.

This analysis could provide useful information for identifying those ruxolitinib-treated patients that are at higher risk of SARS-CoV-2 infection and assessing prognostic factors for survival in a homogeneously treated cohort. The final objective is to provide decision-support tools for viral therapy and/or hospitalization.

## Methods

### Study setting

The observational retrospective cohort studies “RUX-MF and “PV-ARC” were promoted by the IRCCS Azienda Ospedaliero-Universitaria S. Orsola-Malpighi, Bologna, Italy. The PV-ARC study involves 934 PV patients, while the “RUX-MF” study collects 886 MF patients in chronic phase who received ruxolitinib outside clinical trials. Details of protocol design, list of participating Centres and operational procedures have already been reported [8, 9]. For the purposes of this analysis, data concerning MF/PV and characteristics related to first Covid19 infections during ruxolitinib therapy were recorded. The data cut-off date was January 2022.

Waves of the Covid19 pandemic were divided into three periods, according to the type of predominant circulating variants in Europe: first (wild-type variant, February–June 2020); second (alpha/beta/gamma variants, July 2020–June 2021) and third (delta variant, July 2021–January 2022).

Covid19 severity was categorized according to the NIH Guidelines [10].

### Statistical analysis

Statistical analysis was carried out at the biostatistics laboratory of the MPN Unit at the Institute of Hematology “L. and A. Seràgnoli”, IRCCS Azienda Ospedaliero-Universitaria, Bologna.

Continuous variables have been summarized by their median and range, and categorical variables by count and relative frequency (%) of each category. Comparisons of quantitative variables between groups were carried out by Wilcoxon–Mann–Whitney rank-sum test; association between categorical variables was tested by the χ2 test. By Receiver Operating Characteristic (ROC) curve, the optimal cut-off for neutrophil to lymphocyte ratio (NLR) was found at 5.5 (AUC: 0.66) for hospitalization and at 6.8 (AUC: 0.71) for death.

Using Cox proportional hazard model, association with COVID-19 hospitalization and Covid19-related survival was evaluated for the following variables: age ≥ 70 years, sex, presence of at least one comorbidity, MPN type, NLR ≥ 5.5 (hospitalization), NLR ≥ 6.8, vaccination, wave, previous thrombosis, and platelet count/hemoglobin at infection. The same factors were evaluated using a logistic regression model for PV and MF patients (adding DIPSS and spleen response at Covid19 infection in the latter). The association between thromboses that occurred during the pandemic and Covid19 infection, MPN type and NLR was also investigated.

For all analyses, the starting time was February 2020, corresponding to the pandemic start.

Overall survival was calculated by Kaplan–Meier analysis, starting from the date of Covid19 infection and considering only Covid19-related deaths.

Pearson’s test was used to measure the collinearity of covariates.

Akaike’s Information Criterion (AIC) and Schwarz’s Bayesian Information Criterion (BIC) were used to choose the model that best fits the data.

For all tested hypotheses, two-tailed *p*-values < 0.05 were considered significant. Statistical analyses were performed using STATA Software, 15.1 (StataCorp LP, College Station TX, USA).

## Results

### Study cohort

Overall, 886 MF and 172 PV patients treated with ruxolitinib outside clinical trials have been registered in the RUX-MF and in the PV-ARC databases, respectively. At pandemic start, 560 patients (413 MF and 147 PV) were receiving ruxolitinib and were included in this analysis. Ruxolitinib dose was evaluable in 135 and 409 PV and MF patients, respectively. Median dose at pandemic start was 5–10 mg BID in all PV and 189 (46.2%) MF patients, 15 mg BID and 20 mg BID in 114 (27.9%) and 106 (25.9%) MF patients.

From February 2020 to January 2022, 83 (14.2%) patients acquired the Covid19 disease (PV *n* = 16, 10.8%; MF *n* = 67, 16.2%; *p* = 0.12), with an overall incidence rate of 10.5 per 100 patient-years. Overall, 15, 41, and 27 infections were observed during the first, second, and third pandemic wave, with incidence rates of 6.5, 7.8, and 7.3 per 100 patient-years in the three waves, respectively (*p* = 0.75).

Infection was asymptomatic/mild in 21 patients (25.3%), moderate in 17 (20.5%), severe in 18 (21.7%), critical in 6 (7.2%) and fatal in 21 (25.3%) patients (Supplementary Fig. 1).

### Characteristics associated with Covid19 infection and hospitalization

Differences between non-Covid19 and Covid19 ruxolitinib-treated patients are summarized in Table 1. Overall, 371/467 evaluable patients (79.4%) received ≥ 1 dose of anti-SARS-CoV2 vaccine. All but one patient received an mRNA vaccine (BioNTech/Pfizer *n* = 327 [88.1%], Moderna *n* = 43 [11.6%]).

**Table 1:** Patients’ characteristics by SARS-CoV-2 infection and hospitalization.

	Overall cohort (n. 560)			SARS-CoV-2 infected patients (n. 83)		
Characteristics	Non-covid (n. 477)	Covid (n. 83)	*p* value	Non-hospitalized (n. 38)	Hospitalized (n. 45)	*p* value
**Male sex,** ***n*** **(%)**	249 (52.2%)	47 (56.6%)	0.46	20 (52.7%)	27 (60%)	0.50
**Age at pandemic start, median (IQR)**	70.5 (32.9-89.5)	70.5 (46.4–89.4)	0.91	65.3 (46–79.7)	72.7 (52.8–89.4)	**0.005**
≥70 yrs	153 (32.1%)	29 (34.9%)	0.61	9 (23.7%)	20 (44.4%)	**0.05**
**Comorbidities, no. (%)**	170 (35.61%)	23 (27.7%)	0.16	9 (23.7%)	14 (31.11%)	0.45
**MF diagnosis, no. (%)**	346 (72.5%)	67 (80.7%)	0.12	29 (76.3%)	38 (84.4%)	0.35
**Previous thrombosis, no. (% on evaluable)**	81 (20.7%)	8 (13.3%)	0.18	2 (8.70%)	6 (16.22%)	0.41
**Median time from MF/PV diagnosis to SARS-CoV-2 infection, years (range)**	NA	6 (0.4–28.4)	NA	6.9 (0.7–28.4)	5.6 (0.4–21.5)	0.50
**Median RUX duration at SARS-CoV-2 infection, years (range)**	NA	2.8 (0.4–9.9)	NA	3 (0.3–9.9)	2.7 (0.4–8.1)	0.85
**Ruxolitinib discontinuation during SARS-CoV-2 infection, no (%)**	NA	9	NA	0	9 (20%)	0.004
**Ruxolitinib dose, no. (% on 544 evaluable)**			0.56			0.93
5–10 BID	279 (59.9%)	44 (56.4%)		19 (55.8%)	25 (56.8%)	
15–20 BID	187 (40.1%)	34 (43.6%)		15 (44.1%)	19 (43.2%)	
**Chemistry at SARS-CoV-2 infection, median (IQR)**						
Hemoglobin g/dL	NA	10.55 (4.6–15.1)		10.8 (4.6–15.1)	10 (6–13.8)	0.11
Hematocrit %	NA	32.9 (11.7–47.2)		33.9 (11.7–47.2)	31.8 (18.8–46.0)	0.35
WBC ×10^9^/L	NA	7.17 (1.0–115.1)		6.5 (2.6–28.6)	9.0 (1.0–115.1)	0.18
Neutrophils	NA	5.1 (0.8–99)		4.2 (1.7–18.6)	7.2 (0.8–99)	**0.04**
Lymphocytes	NA	1.12 (0.2–25.0)		1.10 (0.4–13.3)	1.15 (0.2–25.0)	0.72
N/L ratio	NA	4.4 (1.4–17.3)		3.5 (1.4–11.9)	5.6 (2.0–17.3)	**0.04**
Platelets ×10^9^/L	NA	183 (2–707)		226 (38–707)	150 (2–487)	**0.02**
**SARS-CoV-2 vaccination before infection, no. (% on 467 evaluable)**	324 (83.5%)	26 (32.9%)	**<0.001**	20 (55.6%)	6 (13.9%)	**<0.001**
**SARS-CoV-2 vaccination dose before infection, no. (% on 346 evaluable)**			0.48			0.24
1 dose	5/320 (1.6%)	1/26 (3.9%)		0 (0%)	1 (14.3%)	
2 doses	88 (27.5%)	9 (34.6%)		7 (36.8%)	2 (28.6%)	
3 doses	227 (70.9%)	16 (61.5%)		12 (63.2%)	4 (61.5%)	

The characters in bold were entered for two different reasons: (1) For *p* values, only statistically significant *p* values have been marked in bold here. (2) For the characteristic names. All 'titles' of the different characteristics have been entered in bold.

Compared to Covid19 patients, those who did not acquire the infection had more frequently received ≥ 1 dose of anti-SARS-Cov2 vaccine (*p* < 0.001). The protective effect of vaccination was confirmed also in the MF and in the PV population separately (39.5% and 56.3% of vaccinated patients vs. 82.5% and 86.8% of unvaccinated patients with Covid19 infection in MF and PV, *p* < 0.001 and *p* = 0.003, respectively).

All the 45 (54.2%) patients with severe, critical, fatal infections were hospitalized. The frequency of hospitalization in the first and second waves (66 and 68%) was higher compared to the third one (26%), (*p* = 0.002). Compared to outpatients, those admitted to hospital were more likely to be ≥ 70 years (*p* = 0.05), had a significantly lower median platelet counts (150 vs. 226 × 10^9^/L, *p* = 0.02) and higher neutrophil counts (7.2 vs. 4.2 × 10^9^/L, *p* = 0.04), with a significant increase of neutrophil to lymphocyte ratio (NLR) (5.6 vs. 3.5, *p* = 0.04). At Covid19 diagnosis, ruxolitinib was reduced in 11 (13.3%) patients. Ruxolitinib discontinuation occurred in 9 patients (10.8%) in the 1st, 2nd and 3 wave in 4, 4 and 1 patients, respectively, and comparably in MF and PV. The cause of discontinuation was severe Covid19 infection in all cases, together with severe thrombocytopenia (platelet < 50 × 10^9^/l) in 2 cases.

**Fig. 1:**
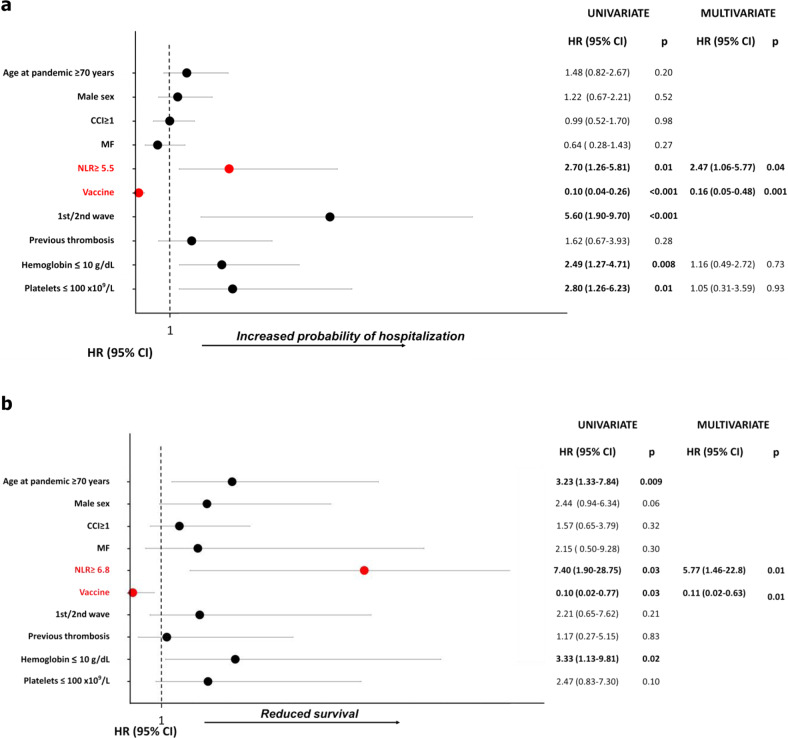
Risk factors. Risk factors associated with hospitalization (**a**) and survival after Covid19 (**b**). Overall, the percentage of patients aged ≥ 70 or with NLR ≥ 5.5 was comparable across the three Covid19 waves. Risk factors for hospitalization and mortality were calculated by Cox proportional hazard model.

In multivariate analysis, NLR ≥ 5.5, (HR[95%CI]: 2.47, [1.06–5.77], *p* = 0.04) and being vaccinated (HR[95%CI]: 0.16 [0.05–0.48], *p* = 0.001) remained associated with increased and reduced risk of hospitalization, respectively (Fig. 1a).

Analyzing MF patients only, hospitalized patients were older (64.5 vs 72.2 years, *p* = 0.02), had a lower median level of hemoglobin (9.9 vs 11 g/dL, *p* = 0.004) and platelet counts (130 vs. 221 × 10^9^/L, *P* = 0.002) and higher neutrophil counts (7.3 vs. 3.8 × 10^9^/L, *p* = 0.04), with a significant increase of NLR (5.6 vs. 3.4, *p* = 0.03). Also, the absence of spleen response and not being vaccinated at Covid19 infection remained significantly associated with hospitalization (OR[95%CI]: 3.38[1.04–11], *p* = 0.04) and (OR[95%CI]: 6.7[1.99–22], *p* = 0.002). PV hospitalized patients presented more frequently comorbidities (57.1% versus 11.1%, *p* = 0.045)

Notably, 12 thromboses (10 venous, 2 arterial) were observed, with an incidence rate (IR) of 1.9 per 100 patient-years. Two venous thromboses occurred in Covid19 patients (all hospitalized) (IR 6.7 per 100 patient-years) and 10 thromboses occurred in non-Covid19 patients (IR 1.7 per 100 patient-years, *p* = 0.05). Out of 505 evaluable patients, 8/384 (2.1%) and 4/121 (3.3%) thromboses occurred in MF and PV patients, respectively (*p* = 0.44). Since NLR has been observed as a novel predictor of venous thrombosis in polycythemia vera [11], we investigated the risk associated with NLR ≥ 5.5 for thrombosis in SARS-CoV-2 infected patients, but no significant association was found, possibly due to small sample size (OR[95%CI]: 0.74[0.63–8.67], *p* = 0.81).

### Characteristics associated with Covid19-related mortality

Overall, 21 patients died due to Covid19 infection, after a median time of 8 days (range, 4–44) from Covid19 diagnosis. All were hospitalized. Among the 9 patients who discontinued ruxolitinib, 5 died (*p* = 0.05) (Supplementary Fig. 2). The frequency of Covid19-related deaths decreased over time, with 46.7% (7/15), 29.3% (12/41) and 7.4% (2/27) of deceased patients in the first, second and third wave, respectively (*p* = 0.01).

In multivariable analysis, probability of survival was significantly lower in patients with NLR ≥ 6.8 (HR[95%CI]: 5.77[1.46–22.80], *p* = 0.01). Conversely, vaccination was associated with reduced risk of death (HR[95%CI]: 0.11[0.02–0.63], *p* = 0.01) (Fig. 1b).

## Discussion

This study provides epidemiological data on Covid19 infection in MPN patients treated with ruxolitinib, showing that 14.2% of such patients acquired the infection, with an incidence rate of 10.5 × 100 patients-years. The incidence did not change significantly in the three waves. This confirms previous reports on MPN patients [12] and probably reflects the rapid administration of vaccines in these oncological patients, with reduced spread of the most infectious variants. Indeed, only vaccination status could significantly reduce the risk of infection in this cohort.

However, among infected and hospitalized patients, 32.9% and 13.9% were vaccinated, respectively. These data are slightly higher than those recently described in a general population of MPNs [12], and are possibly due to a negative impact of ruxolitinib. Conversely, these incidences are superior to those reported in patients with more aggressive hematological neoplasms, in which Covid19 infection was severe in 60.7% of vaccinated patients [13]. A high NLR ratio, suggestive for a high degree of inflammation, was also associated with hospitalization and death, as already noted [14].

In MF patients, a significant association between lack of spleen response to ruxolitinib and increased risk of hospitalization was observed. This data reinforces the protective role of response on outcome [15].

Finally, we confirmed that mortality in patients with MPN and Covid19 is high, particularly in the elderly and unvaccinated and who abruptly discontinued ruxolitinib during the acute phase of infection [5, 1]. These findings were shown in the first wave of pandemic, sustained by the wild-type virus, and declined significantly during the third wave, as already reported [12].

Overall, this analysis highlights that ruxolitinib-treated patients represent a frail cohort with high Covid19-related morbidity and mortality. The absence of vaccination, particularly in patients ≥ 70 years and with high NLR, is associated with severe infection and reduced survival. In MF patients, lack of spleen response to ruxolitinib predicts hospitalization. These features should prompt anti-viral therapy in ruxolitinib-treated patients.

## Supplementary information

Supplementary information

## Data Availability

The datasets generated during and analyzed during the current study are available from the corresponding author on reasonable request.
