# Supplementary material for: Determinants of Covid19 disease and of survival after Covid19 in MPN patients treated with ruxolitinib
Source: Blood Cancer J. 2023 May 3;13(1):65. doi: 10.1038/s41408-023-00834-6 (PMC10155661; doi:10.1038/s41408-023-00834-6)

Supplemental Figure 1. Patient flowchat

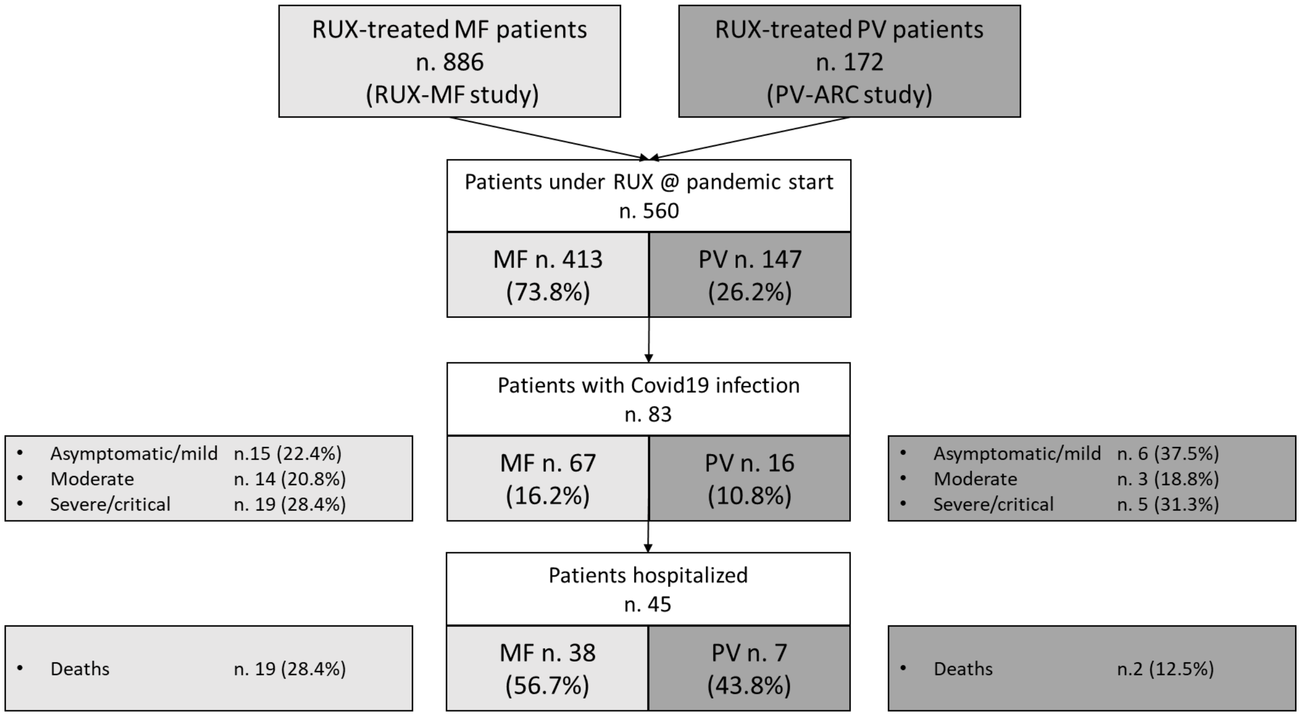

Supplemental Figure 2. Overall survival by Ruxolitinib continuation or discontinuation.
Kaplan-Meier curves of overall survival by Ruxolitinib continuation or discontinuation.

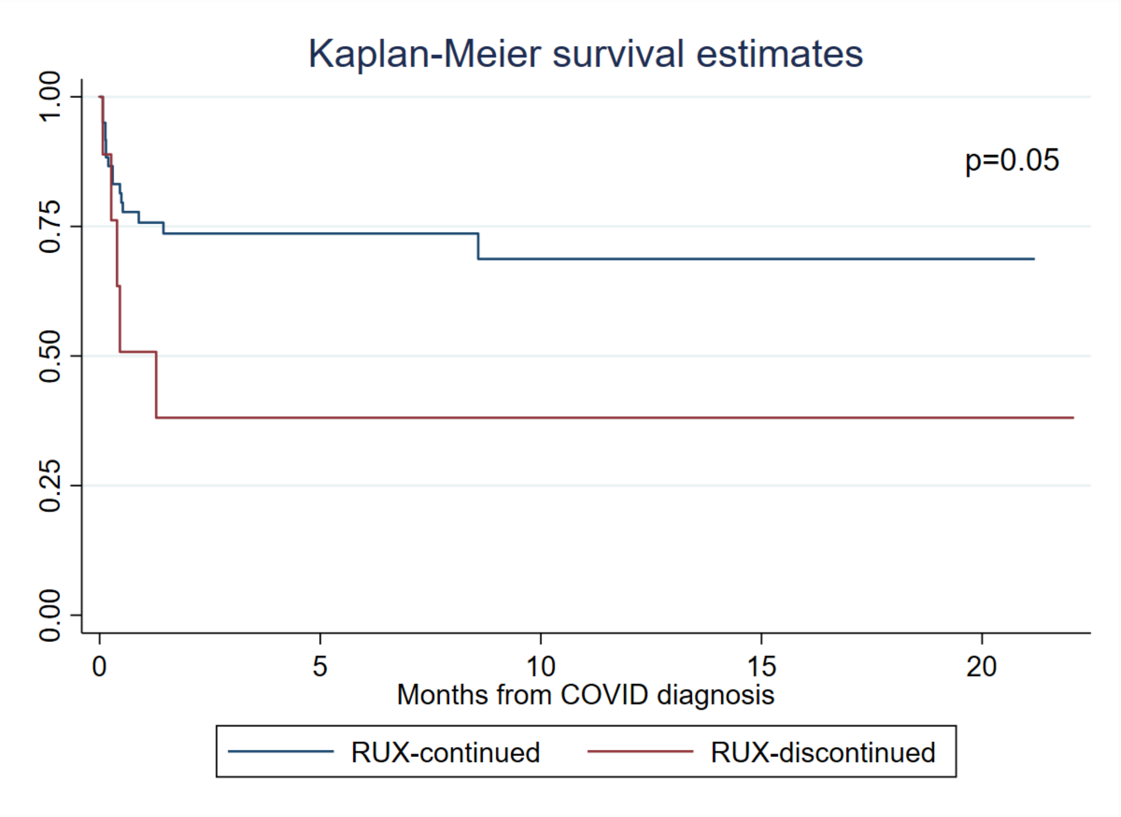

Supplement: Supplementary file 1 — Supplementary information [file 41408_2023_834_MOESM1_ESM.pdf]
